# A Citywide ‘Virus Testing': Chinese Government's Response to Preventing and Controlling the Second Outbreak of SARS-CoV-2

**DOI:** 10.3389/fpubh.2021.601592

**Published:** 2021-06-16

**Authors:** Liting Zhou, Hans Nibshan Seesaghur, Nadeem Akhtar, Jason Boolakee, Cornelius B. Pratt

**Affiliations:** ^1^School of Urban Culture, South China Normal University, Foshan, China; ^2^Faculty of Public Health, University of South Wales, Pontypridd, United Kingdom; ^3^Lew Klein College of Media and Communication, Temple University, Philadelphia, PA, United States

**Keywords:** control mechanism, crisis management, massive virus testing, SARS-CoV-2, Wuhan

## Abstract

Containing the spread of SARS-CoV-2 is a daunting challenge globally. China, as well as a handful of other countries, has, for the most part, contained it by implementing strict policies. Wuhan's citywide virus-testing program presents a way forward in preventing and controlling the uncertainty, anxiety, instability and complexity it faces over the outbreak of SARS-CoV-2. Inarguably, the health crisis requires time-tested strategies and tactics for coordinating governments' and social entities' response to the health crisis, with a goal toward having and ensuring sustained effectiveness. Because of a possible recurrence of SARS-CoV-2 in Wuhan, the Prevention and Control Headquarters of Wuhan on COVID-19 launched a massive virus testing of Wuhan's 11 million residents; it was completed within 10 days. In light of this unprecedented mass testing, this study applies the situational crisis communication theory to analyze this massive virus-testing process and the mechanisms involved to contain SARS-CoV-2 in Wuhan. While many countries still have partial lockdowns, the second outbreak in Wuhan was an indication of what awaited all SARS-CoV-2-stricken countries post-lockdowns and after community restrictions had been lifted. Therefore, the recently implemented Wuhan control mechanism (in cities, districts and townships) may become a hortatory guide to other world regions as they contend with and consider appropriate measures to control the spread of SARS-CoV-2 and to ensure public safety.

## Introduction

The evolving SARS-CoV-2 crisis presents a daunting challenge and a continuing conundrum to the global community (1–6). During the past two decades, the world responded to a series of virulent health crises: SARS, MERS, H1N1, Zika and, recently, SARS-CoV-2, which, to date, has affected more than 190 countries and territories, resulting in more than 170 million confirmed cases and 3.5 million deaths (7). This enormous devastation is mainly due to human behavior and to the response of local and state governments worldwide (8). The coronavirus disease (COVID-19) is mainly caused by a virus named severe acute respiratory syndrome coronavirus 2 (SARS-CoV-2). COVID-19 was so branded officially by the World Health Organization (WHO) on February 11, 2020. Institutions worldwide are continually monitoring the trajectories of coronaviruses and their tendency to trigger new outbreaks. Similarly, governments across the globe have persistently introduced mechanisms and management strategies to cope with a variety of national challenges—from natural disasters to environmental issues to epidemics to pandemics. An effective response has always been a challenge to central, provincial and local governments, as they strive to enhance their public credibility during such emergencies. The degree of complexity and seriousness varies from crisis to crisis and the social governance of governments is expectedly stretched. Generally, an early response through a well-established prevention-and-control mechanism can make a difference in effectively controlling and resolving a crisis.

The Wuhan government has used test-trace-isolation-treatment approach, before going for the citywide mass testing. On May 7, 2020, a male person from Sanmin Community, Changqing Street, Dongxihu District, tested positive for the nucleic acid test. On May 9, after reexamination, the antibody result was positive, and was identified by experts as a confirmed case of SARS-CoV-2. The virus-testing result of his close contact, his wife, was also positive but asymptomatic; their daughter's was negative and she presented no symptoms. There were 20 confirmed cases in the community where the patient lived. Public-health experts analyzed various factors and found that the cause was mainly from past community infections (9). Since the case's positive result was known on May 7, the national and provincial disease control experts immediately conducted epidemiological investigations and administered virus tests on community residents. Five asymptomatic cases were recorded. By adopting the situational crisis communication management strategy, the leaders of the provincial, municipal, and district governments attached great importance to the epidemic and quickly adopted several prevention and control measures. First, they implemented centralized isolation and nucleic acid testing, for key populations and close contacts, and carried out traditional Chinese-medicine intervention. Second, they decided to lock down the community while increasing environmental governance and disinfection, as this strategy is considered one of the effective ones (10). Third, they made every effort to strengthen treatment and transfer all asymptomatic cases to hospitals for medical observation and combined Chinese and Western medical treatment.

The six new confirmed cases of SARS-CoV-2 in Sanmin ended Hubei province's status of having no new confirmed cases in 35 consecutive days. To prevent a possible outbreak and to screen for potential asymptomatic infections in communities such as Sanmin, the Prevention and Control Headquarters of Wuhan on COVID-19 took prompt actions, guided by the possibility of a second surge in SARS-CoV-2 cases. An “Emergency Notice on Launching the virus testing of SARS-CoV-2 for the Entire City” was issued May 11 to tackle the possible threat and to target residents for citywide virus testing within 10 days. The ultimate objective of this massive scale of testing was to target untested residents from both dense and remote areas of Wuhan. In light of this initiative, the Wuhan Municipal Government convened a phalanx of experts for frequent coordination briefings during the mass testing and screening of asymptomatic infected people in Wuhan. Further, it was decided that the scope of virus assay would be expanded. Virus testing for all citizens would be carried out and a comprehensive screening of asymptomatic infected people would also be made, based on the previous three million virus assays that were carried out during the first wave of the pandemic. For such an operation to become realizable, funds were disbursed by both the municipal and district finance in a 1:1 proportion (11). Currently, the demographic data indicate that the resident population of Wuhan is 10.89 million (12). Based on that calculation, there are still about 7.89 million people waiting to be tested. During the first wave of the outbreak, three million were tested; they were excluded from the second-wave virus testing. The Hubei provincial government immediately made 53 virus-testing institutions and 211 virus-testing sites in Wuhan available to residents, for an average daily detection capacity of 46,000 people (13). The 10-day timeframe implied that a daily average detection capacity of about 80,000 people would be needed. Ten additional virus-assay institutions for COVID-19 were established (14) to meet the daily detection capacity. Thus, the Municipal Health Commission applied all of its resources in its effort to reach the set target, thereby ensuring its strategic effectiveness in containing the second surge of SARS-CoV-2 in Wuhan.

In view of the still-severe situation of global epidemic prevention and control, how does the Wuhan Municipal Government assuage public concerns and sensitivities, engage in credible publicity and in information dissemination on virus testing, and encourage voluntarily participation in the testing? How do the local government's persuasive messages ensure that the work can be completed successfully in the shortest possible time? What is the effect of hierarchical prevention and control and social linkage emergency management mode adopted by local governments in response to public-health emergencies? How and why can Chinese local governments achieve the prevention and control of the pandemic through such top-down social mobilization? The answers to those questions underpin the objectives of this article, in hopes of providing a government-level point of reference on extant research literature on SARS-CoV-2 prevention and control. The rest of this article is organized into five main sections: (a) review of the literature, followed by our two-pronged theoretical framework; (b) methods and strategies; (c) results; (d) discussion; and (e) implications of the theoretical framework for governments' pandemic response. The conclusion section asserts the public-health resolve of the Wuhan government to protect its citizens from the devastating effects of an impending SARS-CoV-2.

## Literature Review

The extant research on SARS-CoV-2 was conducted primarily in fields such as medical science, public health, informatics, and communication, but rarely from the perspective of public-health administration and its response to unconventional and unprecedented public-health emergencies. Even though studies have been conducted from a crisis-management perspective, they focused on the early phase of SARS-CoV-2outbreak (15, 16). The present study, however, focuses on the upcoming crisis of SARS-CoV-2. Research on prevention and control mechanisms of local governments' responses to epidemics in China and on their specific response to them is also minuscule. The current pandemic still wreaks havoc in communities, urban and rural, making the rationale for this study on the emergency-management response of local governments in China a point of departure for other governments, even as this study also provides comparative materials on COVID-19 prevention and control for public-health academics.

### Studies on COVID-19 in General and Virus-Testing Approaches

Research on COVID-19 can be presented in three broad categories: (a) research published by medical professionals and experts to highlight its genetic makeup, pathophysiological manifestations, associated signs and symptoms, its incubation process and complications (17–19); (b) research that focuses on the preparedness and risk management at the organizational level, specifically addressing the prevention and control measures for frontline health professionals (20–22); and (c) reviews that highlight the general impact of COVID-19 on countries' socioeconomic environment and their responses to the pandemic (23–26).

Abrams and Greenhawt (26) noted that effective risk communication could keep patients and the public well-informed. Zhou, Su and Pei et al. (27) noted that the geographic information system has played a crucial role in fighting against the pandemic by tracking the confirmed cases and providing information on risk and prevention. The fast information-dissemination system provided quick information on safety and prevention measures, besides lockdowns.

Virus testing, a research category in its own right, offers more sensitive and early detection of SARS-CoV-2. The extant literature identified a variety of options for fast, cost-effective and sensitive virus testing and virus testing was suggested as the most reliable approach for early assay of SARS-CoV-2 (26–28). Esbin, Whitney, Chong et al. (28) categorized testing methods into two: viral and serological. In the former, the RNA virus collected from a patient's throat or nasal passage is directly examined, whereas the serological test detects antibodies in the patient's serum (29). Virus testing is most effective in early assay of SARS-CoV-2; hence, it explains the massive virus testing of Wuhan residents to contain the further spread of a second-wave of the epidemic in the aftermath of diagnosing six new cases. Before we present the materials and method of this article, we shall provide highlights of its theoretical underpinnings.

### The Theoretical Framework of Crisis Management and Coordination

Based on extant knowledge of the origins of SARS-CoV-2, we conclude that it falls squarely in Ulmer, Sellnow and Seeger's (30) category of an unintentional crisis or Coombs's accidental crisis, both of which “tend to be outside the realm of intentional human provocation” (10, 31). The novel coronavirus was, as far as current clinical evidence suggests, not the doing of any government or of any agency. As Pratt (31) notes, “Crisis communication managers have at their disposal a number of theories that can provide the guiding light to what such response *should* be” (10). Therefore, Coombs's situational crisis communication theory (SCCT) (32, 33) provides a road map for Wuhan government's provincial response to SARS-CoV-2. Cast against that backdrop, SCCT is guided by answers to a two-pronged question: (a) How do Hubei residents and the world at large respond to the ensuing health crisis? (b) What are the best response strategies the Wuhan local government can adopt to restore public confidence and its public reputation? The audience-centered theory is grounded in attribution theory; therefore, the more the cause of the crisis is viewed publicly as beyond the control of Wuhan local government, the lower the attribution of responsibility to the government.

McConnell (34) rightly explained the success and failure of crisis management by suggesting that “crisis management initiative is successful only if it follows pre-anticipated and/or relevant processes and involves the taking of decisions which have the effect of minimizing loss of life/damage, restoring order and achieving political goals, while attracting universal or near universal support and/no or virtually no opposition.” Crisis management is fraught with uncertainty, instability and complexity, thus requiring a specific approach to ensuring coordination between government institutions and social entities and increasing the efficiency of the governance system and of crisis-management performance (35). In that context, Christensen and Ma (35) noted categorically that “without strong coordination capacity to mobilize various entities to respond concertedly to emergencies, crisis management can neither succeed nor sustained.” In other words, coordination is key to bringing all the interdependent actors into active interaction to respond effectively to a crisis.

This study also subscribes to Christensen and Ma's (35) perspective on coordination, which was specifically developed in the Chinese context. They presented coping strategies in crisis management for analyzing measures taken by the Wuhan government to contain further spread, by introducing citywide virus testing of all Wuhan residents. The framework of coordination, vertical and horizontal coordination in crisis management posits that, in the upper-right quadrant, both horizontal and vertical coordination mechanisms are in high gear to respond this ongoing health crisis. Based on the Christensen and Ma's (35) theoretical framework, we may induce that the relevant ministries may simultaneously coordinate with local government institutions while responding to the second-wave of SARS-CoV-2. Indeed, such a massive move, to test 6–8 million people in 10 days, requires a well-established joint coordination mechanism among different local government and social institutions to make their crisis management efforts a success.

## Methods and Strategies

### Specific Implementation of Virus Testing in Wuhan

#### Organizational Structure and Control Mechanism

According to the requirements of the *Emergency Notice* issued by the Prevention and Control Headquarters of Wuhan on COVID-19, to cover the entire city within the 10-day timeframe, the virus assay was implemented in phases, districtwide. The first step required the district government to normalize the requirements of epidemic prevention and control according to the population scale, and to formulate the whole virus screening plan in combination with the actual situation of the district. That means there is a strong need to develop active and close coordination among different local institutions and administrative units to implement the virus-testing plan effectively. It requires an immediate mobilization of relevant resources such as reagents, equipment and trained medical and para-medical staff to ensure the maximum testing of the residents to meet the target timeframe.

**Figure 1:**
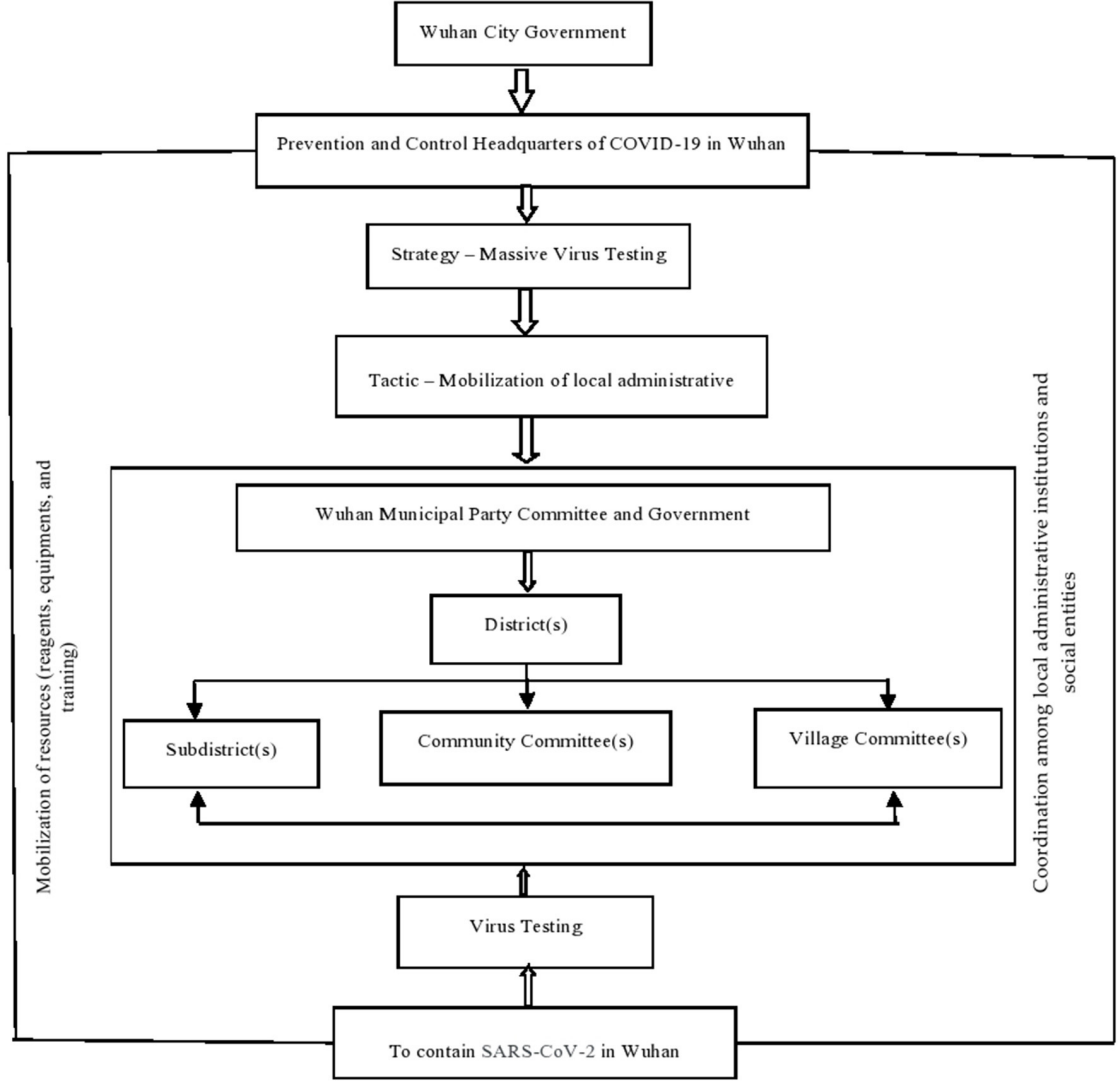
Framing of control and coordination mechanism by applying the situational crisis communication theory to conduct virus testing in Wuhan.

The aim of this article is to analyze the implementation of Wuhan's citywide virus-testing program. But the impact of the pandemic, restrictions on local movements and 14-day isolation measures issued by governments across China translate into the authors not being able to immerse themselves in Wuhan government departments and in specific communities to conduct necessary field investigations. Therefore, there is a palpable lack of firsthand data. Because this study is based entirely on official government reports, it does not have a comprehensive methodology. For example, it lacks key data on the number of staff involved in the large-scale virus testing, the intensive training of staff, and the specific logistical support measures of governments. The authors developed a control mechanism chart by applying the coordination theoretical framework to work at both vertical and horizontal dimensions to ensure the effectiveness of the crisis management (Figure 1).

Figure 1 shows a clear trajectory of the Wuhan Municipal Government, which implemented a citywide virus testing to reduce a further spread of the epidemic and eliminate potential risks. The local government first builds a broad social consensus and makes SARS-CoV-2 prevention and controls its highest priority, then sets up the epidemic prevention and control headquarters and, in sequential levels, implements it in the corresponding district, township, street or community management departments. This shows that at the government level, the society has moved from a normal state to a state of emergency. In addition to working through the organizational structure and enabling the rapid start and high-speed operation of information and resources, the government also makes it possible to maximize social mobilization and carry out “Movement Governance.” In short, under this kind of mechanism, it is very beneficial to focus attention, work through conventions, concentrate resources to execute the prevention-and-control model of “Holistic Government,” and conduct blanket virus testing. The government's organizational setup for addressing pandemics leads to two structural observations.

First, from a vertical perspective, under a strong accountability system, epidemic prevention and control have become the main indicators for evaluating the effectiveness of local government governance. This figure can, therefore, facilitate the implementation flow of government orders from top to bottom, ensuring grassroots inclusivity in the process, and providing a mechanism guarantee for local officials to mobilize various resources to the greatest extent to carry out prevention and control. Second, from a horizontal perspective, the implementation of citywide virus testing in Wuhan, a high-risk area, not only requires government departments not only to deploy resources, but also requires of them extensive mobilization of enterprises, nongovernmental organizations, and the engagement of the public in participating fully in the process of epidemic management. It must be noted that the unified thinking and collectivist values in traditional Chinese culture have helped reduce the social cost of epidemic control and coordination. The Chinese tend to put national interest first and are willing to give up part of their personal interests, rights, or spaces so that local governments confront less resistance in implementing a virus-testing policy, a subject to which we shall now direct our attention.

#### Administrative Divisions and Testing Focus

Under the joint leadership of the street and community party organizations, all communities, together with institutions within their jurisdiction, such as the residents committee, the owners committee, the property management company, the community health service center, the community police office, volunteer groups and families, jointly assisted in the large-scale testing, forming a multiparty collaborative governance mechanism at the grassroots level. The district government needed to further clarify the basic situation (population base, number of streets, number of communities and their locations), organizations, time arrangement (specific to the sampling time of streets and communities) and other precautions. Wuhan includes 17 administrative districts (functional areas). Each district has 156 subdistrict offices, one town and three townships, 1,337 community committees and 1,814 village committees among them (36), as presented in Table 1.

**Table 1:** Administrative divisions, resident population and grassroots units of Wuhan.

**District**	**Resident population (10,000 people)**	**Subdistrict offices (unit)**	**Community committees (unit)**	**Village committees (unit)**	**Town governments (unit)**	**Township governments (unit)**
Whole City	1089.29	156	1,337	1,814	1	3
Jiang'an	96.24	16	142	14		
Jianghan	72.96	13	108	0		
Qiaokou	86.85	11	127	1		
Hanyang	65.27	11	118	0		
Wuchang	125.86	14	140	1		
Qingshan	52.88	10	83	12		
Hongshan	117.16	13	161	3		1
Caidian	45.93	11	46	283		1
Jiangxia	70.51	15	66	268		
Huangpi	98.83	15	77	589		1
Xinzhou	90.21	12	67	546	1	
Dongxihu	56.25	11	65	0		
Hanan	13.55	4	15	29		
Wuhan economic and technological development zone	27.57		37	21		
East lake high-tech development zone	56.14		68	26		
East lake ecotourism scenic zone	8.46		16	1		
Wuhan chemical industry park	4.62		1	20		

*Wuhan Statistical Yearbook, 2018, Wuhan Bureau of Statistics*.

In sum, new confirmed cases districtwide, as of May 14, 2020 (that is, before the start of large-scale testing), totaled 50,339 in Wuhan. They ranged from 483 cases in Donghu Scenic Zone to 6,563 in Jiang'an District to 7,551 in Wuchang District (37). The focus was fourfold: (a) identifying the detection range, (b) targeting the priority-detection population, (c) identifying further the number of asymptomatic infections, and (d) implementing effective controls. More emphasis was placed on priority detection groups: old urban areas, communities with dense external tenants, urban-rural junctions and residential areas around large markets. In places where positive or asymptomatic infections were found, the scope of nucleic acid testing was expanded to improve its efficiency. However, it must be noted that children younger than 6 years were excluded, even though their numbers were not released.

Using Jiang'an District as an example, by widely depending on the advice of field and community representatives on epidemic prevention and control, the district headquarters issued on May 12 the “Notice on Deepening the Party Construction and Leading the Grassroots Governance for an Effective Approach to Community Epidemic Prevention and Control.” This notice implied that the degree of community epidemic prevention and control would be improved comprehensively through strengthening the subdistrict working committee and community party committee, establishing temporary party branch as check-points, implementing the community (grid) party organization, and enriching the building party group and party-member central household, while reducing significantly ground-level inanition. Moreover, the normalization guarantee mechanism would be adhered to, and efforts would be made to establish corresponding normal guarantee mechanism from the aspects of human resources, material resources, systems and incentives (38).

## The Testing Process: Analysis in Wuhan

### Nucleic Acid Test Rationale and Laboratory Practice

The Nucleic Acid Test (NAT) is regarded as an effective and immediate method of detecting the SARS-CoV-2 virus because of its high degree of sensitivity and specificity. The rationale behind this test is that nucleic acids, which are essential biomolecules, are present in large amount in all living things. The nucleic acids or bio-polymers are commonly known as the DNA or RNA of a molecule and encode all the genetic information of a particular organism. The nucleic acid test is a technique for detecting the genomic sequence, which helps to identify different species, usually pathogenic microorganisms, such as bacteria or viruses in blood. NAT works by amplifying those genetic strands through a process known as polymerase chain reaction (PCR) which are then paired with the RNA specific to the corona virus for detection. By identifying the genetic components rather than detection of antigens or antibodies from blood samples that require time to appear, health-care providers have an upper hand in early diagnosis of COVID-19. The sample from which the genetic materials are extracted is taken from nasopharyngeal swab or sputum of patients being tested. Those samples have to be handled with maximum care to avoid any contamination of the laboratory staffs by observing very strict sterilization protocols and wearing protective equipment. In fact, for molecular analysis of the pathogen to take place, it is important that the inactivation process of the molecule guarantees a complete loss of the infectiousness of the virus, but at the same time, protects the integrity of the nucleic acids. Only the inactivated samples are safe to be manipulated for testing. Inactivation of the sample involves using extreme *in-vitro* denaturing conditions, mostly in heat, to disrupt the viral envelope and eliminate cellular nucleases. However, the structure of the RNA is preserved for subsequent analyses. Those samples are kept in a highly decontaminated area, under biosafety level 3 standards for diagnosis and later disposed of in sanitary landfills after strict sterilization.

For instance, in a particular virus-detection laboratory in Wuhan constructed in accordance with P2 standards and with a biosafety level 3, and which is also a designated government COVID-19 test base in Hubei province, the samples that came for inspection were packed in a foam box. The sample delivery point was sprayed regularly with alcohol; the foam box was then sent to the inactivated room through a transfer window. The inactivation process prevented the spread of the disease. The technician unpacked the box in a biological safety cabinet in which each sample was handled separately for safety reasons. Samples were then sent to the information review room for accuracy check, using a barcode, and then sealed in a Ziploc bag to reduced probability of contamination. Strict safety management was also reflected during trans-shipment as the outer packaging of the sample was wrapped in an additional plastic container in order to keep the sample fix during transportation. Further, the laboratory staff responsible for the handling of those samples would use a 75% medical alcohol to sterilize both the handle and platform of the transfer window before putting samples in the window.

The testing laboratories operate under strict adherence to guidelines issued by the National Health Commission for biological safety protection and experimental operation. According to the “Laboratory Testing Technical Guidelines of COVID-19 (Second Edition)” and “SARS-CoV-2 Laboratory Biosafety Guidelines (Second Edition),” those laboratories operate using real-time fluorescent quantitative RT-PCR (Re-verse transcription-PCR) technology for RNA extraction and amplification detection. The coronavirus SARS-CoV-2 genome is made up of a leader sequence, ORF1ab which encodes proteins for RNA replication and genes both for non-structural proteins (nps) and structural proteins. The SARS-CoV-2, similar to other beta-coronaviruses, encodes four major structural proteins; namely, the spike (S), envelope (E), membrane protein (M), and nucleoprotein (N). According to the instructions of the nucleic acid kit, the three targets in the virus, namely, the envelope (E), RNA dependent RNA polymerase (RdRP) and the nucleoplasmid (N) genes, are taken as the test objects, and Ct/Cq = 43 as the baseline for result interpretation (negative, single positive, positive): when Ct/Cq ≤ 43 and the amplification curve is typical S type, it is positive.

### The Key Nodes of Virus Testing in Wuhan

#### Qualifications of Nucleic Acid-Testing Institutions and Inspectors

According to the *Laboratory Testing Technical Guidelines on SARS-CoV-2* (Fifth Edition), *Medical Institutions Clinical Gene Amplification Management Measures, Pathogenic Microbial Laboratory Biosafety Management Regulations* and other policy documents, through qualification review and on-site assessment, only qualified professional institutions approved by the provincial headquarters of COVID-19 Epidemic Prevention and Control can carry out testing. Since the SARS-CoV-2 is managed in accordance with the second category of pathogenic microorganisms, the approved institutions have been registered for biosafety on the second-level laboratory and have a reserve of biosafety on third-level laboratory protective equipment. For inspectors, they must obtain a biosafety training certificate for pathogenic microorganisms' laboratory at the municipal level or above, a certificate for clinical gene amplification testing technicians, and a SARS-CoV-2 virus testing.

#### Guarantee of Virus-Sampling Quality

To guarantee the quality of on-site sampling, community-health service centers (township hospitals) and hospitals would be in charge of the sampling work. It was determined by the city government that technical training would be provided to the assistance personnel from these units on how to conduct population sampling. After mastering that technique, personnel will learn the operation of technical instruments required for getting samples, the standard collection methods of nasopharyngeal swab and throat swabs, a sampling team would be organized to sample from each screening unit. Generally, through the method of mixed sample pool (5–10 samples in a group), the slight loss of “specificity” is ignored, and its high “sensitivity” can be used to screen asymptomatic infections on a large scale and greatly improve the testing speed. The municipal and district governments also set up several technical guidance and quality control working groups to ensure the sample quality and testing quality, and to safeguard the precision and reliability of testing results through on-site guidance, supervision and inspection.

#### Registration Form for Testing the People

A registration form was designed to ensure that the tests carried out would be ethically acceptable by the people. Community workers would then send out the notice of free virus screening for all residents online and offline, then organize residents' registration by WeChat group, telephone and household entry, and communicate to them where arrangements for detection points in the community would be made. The community staffs and volunteers allowed the people to register through three channels: (a) advertisements in WeChat grid groups, and build up a sequence for registration; (b) calls to residents separated from their families and live in rented houses to check and explain the intentions behind detection; (c) registrations of the elderly, who are not familiar with the use of smart phones or WeChat and who are disabled or have limited mobility.

#### Strategies for On-Site Sampling

The community was taken as a unit and then divided into districts and buildings to prevent crowding at one place on a short notice. Through micro neighborhood applet, community broadcasting and other means of relaying information, people were reminded about good personal protective measures that they needed to follow. Sampling points in the open area of the community were selected in a manner consistent with ensuring proper sanitation practices like using sanitizers and hand wash, and having proper aeration in public areas. Temperature monitoring points were also set up at the entrance, and special personnel arranged to help residents keep social distance by constantly informing them about its importance. The sampling staffs were also well-protected and made every effort to remind the residents to wear masks (wear one, prepare one). After taking a sample, the gloves would be changed or disinfected. Additional staffs and volunteers were deployed at each sampling point to guide the site protection, handle accidents promptly and ensure the orderly development of the virus testing.

#### Nucleic Acid-Testing Technique and Quality Assurance

The nucleic-acid testing of RT-PCR is the main method for the diagnosis of COVID-19. The SARS-CoV-2 nucleic acid gene is used to design primers for target detection. The nucleic acid detection process of SARS-CoV-2 includes sample collection, storage, transportation, sample pre-processing, nucleic acid extraction, and nucleic acid amplification and detection and result interpretation. To confirm that a case is positive, the test results of at least 2 targets (ORF1ab/N/E) of the SARS-CoV-2 in the same specimen must be positive. In addition, negative results need to exclude factors that may cause false negatives. In order to achieve the purpose of early screening, diversion of suspected cases, timely isolation and treatment of patients, in addition to pathogenic testing and diagnosis, auxiliary diagnosis can be made based on clinical symptoms and CT images.

#### Test Results Query and Privacy Protection

Generally, virus testing results are collected by a special system for storage 24 h after assay. The system is strictly independent, with highly secured login access. The user input is closely monitored to ensure that the personal information security of the inspected citizens is valid and well-protected. If a test result is positive, the relevant department personnel will contact the patient immediately to conduct standardized treatment according to diagnosis and control requirements. If the patient does not receive the relevant notice, the test result will be automatically deemed negative. In addition, residents who have participated in the centralized virus testing can enter the WeChat applet of “Wuhan against the epidemic” and view the result under the “Hubei health code”; or log in the “Official WeChat of health Wuhan” applet by mobile phone and click “Virus result query” to complete the personal identity information and then query the nucleic acid test results. Virus-testing results have indicated, generally, four types of contents to protect the personal privacy: (a) the name of the sample sending organization; (b) sampling time; (c) sample code; and (d) the test results to ensure the security of the personal information of the residents.

## Results

### Effectiveness of the Massive Virus Testing

The main target of the large-scale campaign in Wuhan is focused on permanent and temporary residents who have not been previously subjected to such detections. And the purpose is to find the maximum number of asymptomatic infected people so as to achieve the goal of blocking the spread of the virus from any probable sources. This enables early detection and treatment of confirmed cases, and early isolation of asymptomatic infections, resulting in a safe and healthy environment conducive to the resumption of work and school.

### Numbers and Characteristics of Asymptomatic Infections

According to statistics from Wuhan Health Commission, a total of 665 asymptomatic infections were found in Wuhan since it was “unlocked” on April 8. Some 2,508 close contacts were tracked, of which two were asymptomatic infections; the ratio was 0.8%0, and noconfirmed case was found. As of May 13, in addition to the six confirmed cases in Sanmin, there were 659 asymptomatic infections in total. Based on serological test results, personal trajectories, and previous symptoms of asymptomatic infections, Wuhan CDC determined that 559 of them had previous infections, accounting for 84.8%, while 97 were hidden infections, accounting for 14.7%; three were unclear (39). From the daily epidemic prevention and control dynamics of Wuhan Municipal Health Commission, since the citywide virus assay was officially launched on May 15, Wuhan's single-day capacity of virus assay has been continuously improved until May 23, 2020 (Table 2). Through May 23, the government released daily data while on May 24, in accordance with final data released by the government, more than nine million samples and more than 6.5 million inspections had been completed. It must be noted here that the authors could not find single-day data for May 24. Through May 23, 206 new asymptomatic infections were identified while the final released data showed 218 asymptomatic infections. The detection rate was lower than 0.3 cases per 10,000 people (40).

**Table 2:** Daily virus testing and epidemic situation in each administrative district of Wuhan.

**Date**	**Total number of nucleic acid tests**	**Newly confirmed cases**	**Newly discharged cases**	**New deaths**	**Newly suspected cases**	**New asymptomatic infections**
12/05	42,618	0	0	0	0	6
13/05	67,026	0	0	0	0	11
14/05	72,791	0	0	0	0	9
15/05	113,609	0	0	0	0	9
16/05	222,675	0	0	0	0	10
17/05	335,887	0	0	0	0	14
18/05	467,847	1	0	0	0	16
19/05	856,128	0	0	0	0	13
20/05	887,321	0	0	0	0	28
21/05	1,000,729	0	0	0	0	35
22/05	1,470,950	0	0	0	0	25
23/05	1,146,156	0	0	0	0	30

*Wuhan municipal health commission (the epidemic situation of COVID-19 in Wuhan)*.

It can be inferred from the above data that, because the city undertook the virus assay, as Table 2 shows, asymptomatic infections increased daily. According to the analysis of experts (e.g., Zijian Feng, Deputy Director of China CDC), there are three categories of asymptomatic patients: (a) those with recessive infection, presenting with low infectivity; (b) those with previous infection, presenting with low infectivity, may be due to the presence of antibodies; and (c) those in the incubation period with infectivity. At present, a majority of asymptomatic infections in Wuhan fall into the first two categories, with low infectivity.

### Factors Associated With the Successful Implementation of Massive Virus Testing

In addition, Wuhan Health Commission announced (41), more than 50,000 medical staff and more than 280,000 community workers participated in this large-scale testing campaign. By adding 40 medical institutions and CDCs for testing, there are 63 institutions analyzing the test samples, which greatly increased the overall testing capabilities. These testing agencies also mobilized personnel and added equipment from all over the country. The number of staff in testing institutions increased from 419 to 1,032, and testing equipment increased by 215 pieces to 701. Through these safeguards, the daily testing capacity of Wuhan has increased from 300,000 to more than 1 million. However, it is particularly important to implement a program that eliminates social apprehensions, test hesitancy and prejudice; to promote the overall recovery of the economy and society; and to achieve accurate prevention and control results from testing for the virus in the entire city.

## Discussion

The study raises a crucial issue—the potential threats of a second surge of SARS-CoV-2 and the local governments' response to containing it. The Wuhan government has contained the SARS-CoV-2 outbreak. From March 18 to May 8, 2020, there were no new confirmed cases. Six new cases confirmed on May 9, 2020, in Wuhan, again raised the alarm for the government. The clustering epidemic in the Dongxihu district of Wuhan at the beginning of May suggested that the virus transmission was not contained during the first phase of the SARS-CoV-2 outbreak in Wuhan. Therefore, the Wuhan government decided to launch a citywide nucleic acid testing to ensure the public health safety and to contain the second surge of SARS-CoV-2 effectively. The main purpose of a virus assay was to identify people who carry the virus and to trace their close contacts. One reason for Wuhan's virus-testing program might be its large population in which many people could be (potential) carriers. In other words, to identify the confirmed positive cases and trace people in an urban city are challenging. That was why the Wuhan government launched a citywide-testing campaign.

Indeed, this was also an opportunity for the government to enhance its public image and credibility in a health crisis. Without mobilizing all possible resources, it might not be possible to carry out the citywide testing of about 6.9 million people in 10 days. To the degree that the world is still prone to SARS-CoV-2, the citywide nucleic acid testing by Wuhan's government provides an effective health model. In mid-June, 2020, Beijing Municipal government also tackled the second surge of SARS-CoV-2 with a partial lockdown in which high- and medium-risk areas were closed immediately and residents required to take nucleic acid tests if they thought they were infected. The Beijing Municipal government traced the source of the infection to Xinfadi Food Market; there were no family clusters or cross-infections. To contain the second surge of SARS-CoV-2 in the capital is mainly because of its timely early tracing (42). Similarly, Shanghai government also has the daily capacity of 70,000 tests for SARS-CoV-2, which increased to 90,000 by August 2020 (42). The three city governments' approaches indicate that China has developed a strategic plan for citywide virus testing and also to follow the trace and isolation policy, simultaneously, to prevent a new outbreak of SARS-CoV-2.

Virus assay is recognized as the “gold standard” for diagnosing the new coronavirus infection. For the virus-testing technology and quality assurance, the virus testing of RT-PCR is the main method for the diagnosis of SARS-CoV-2. The SARS-CoV-2 virus gene is used to design primers for target detection. The virus assay process of SARS-CoV-2 includes sample collection, storage, transportation, sample pre-processing virus extraction, virus amplification and detection and the interpretation of results. To confirm that a case is positive, the test results of at least two targets (ORF1ab/N/E) of the SARS-CoV-2 in the same specimen must be positive. In addition, negative results need to exclude factors that may cause false negatives. To achieve the goal of early screening, diversion of suspected cases, timely isolation and treatment of patients, in addition to pathogenic testing and diagnosis, auxiliary diagnosis can be made based on clinical symptoms and CT images.

At the outbreak of SARS-CoV-2, WHO regarded the detection of every suspected case as top priority for epidemic prevention and control. More and more countries now acknowledge the importance of virus assay and are being more compliant with expanding the scope of the test. The Central Leading Group for Responding to the Epidemic of COVID-19 in China held a meeting that called for “accelerating the improvement of detection capacity, carrying out large-scale virus”, and stressed that “this is not only conducive to precise prevention and control, but also conducive to the reasonable flow of personnel, and promoting comprehensive resumption of work and production” (43). The Joint Prevention and Control Mechanism of the State Council also issued the guiding principles on effectively preventing and controlling the pandemic normalization, pointing out that the efficacy of virus assays should be improved, the scope of test expanded, and the key populations tested (44). Wuhan implemented citywide nucleic acid testing to detect confirmed cases and asymptomatic infected people, and to identify and control the sources of infection. It is direly need to ensure the public health and to bring the routine business back to life.

Because the guiding principles issued at the highest level of the Chinese government link results of epidemic prevention and control in various cities to the performance evaluation of local officials, lower-level government agencies and their leadership are encouraged to implement strict prevention measures without the distraction of other concerns, such as the demands of the 10-day nucleic acid testing campaign. This large-scale test can detect confirmed cases and asymptomatic infected people, identify and control the source of infection, thus eliminating transmission channels, minimizing the risk of transmission, and reducing the burden of the disease to the whole society. In the case of strict prevention and defense against the epidemic, China's response was to identify one case and to respond to it head-on. The strategic response here can be enabled by the SCCT to identify key loopholes that may have triggered the second surge of the SARS-CoV-2 crisis, then mobilize all possible resources from the physical to the human to respond effectively. SCCT is appropriate in this context in that it matches crisis-response strategies to a specific crisis. China has used that strategy since Day 1 to contain the crisis; it continues to do so for subsequent surges in Wuhan, Guangzhou, Beijing and Hong Kong.

From the blanket virus assay event in Wuhan, the key to the successful implementation of this impossible task lies in the seamless connection and close cooperation of the three-level government system: city, district and community. In addition, there is continuing need for more social publicity and mobilization. In other words, to restart the daily production and life safely, the government plays a leading role in the epidemic prevention and control, not only playing the role of “Night Watcher” proposed by Adam Smith, but also playing the role of “Visible Hand.” The governments should use public-service announcements and similar forms of publicity to make the citizens informed about and be persuaded to accept the value and importance of testing, and to reduce significantly individual or family anxieties. Also, with the help of the internet and big-data technology, no effort should be spared in reaching the whole staff-detection target. The amplification of virus-assay institutions and the adoption of scientific methods can ensure detection quality while improving efficiency as much as possible. With the continuous global spread and worsening of the pandemic, the role of a responsive government has become increasingly prominent. Wuhan's citywide virus assay reflects the advantages and effects of this normalized prevention and control mechanism, which is enforced by the government, linked up and down, and implemented at all levels.

It must be noted here that through the 10-day nucleic acid-testing competition in Wuhan, the shortcomings of China's public-health emergency management system were gradually revealed. Therefore, it may be useful to reform it in four ways: (a) collect information in various ways to improve the system's risk monitoring and early warning capabilities; (b) improve further the direct reporting system to facilitate the timely upload of information and reduce the tedious process of administrative intervention; and (c) strengthen the ability of scientific analysis, ensuring that the assessment of diseases not be mixed with the concept of administrative hierarchy. When encountering difficult problems, governments at all levels should organize a team of experts to conduct scientific research of the epidemic. The fourth method is to link public health with national security by increasing investment in public health, and strengthening the development of public health personnel. In addition, the epidemic also reveals that China's NGOs still fulfill secondary and passive roles in the prevention-and-control process. Without strong leadership and organization of the government, and strict joint prevention-and-control measures, this competition is unlikely to achieve the expected results in a short time. Therefore, improving the status and the role of NGOs in disease prevention and control, and expanding the breadth and depth of social participation are also key issues that need to be considered by government officials.

## Implications of SCCT for Governments' Pandemic Response

It is important to state here that, because communication practitioners have oftentimes been ill-equipped to apply known theories to resolving crises, there exists a gap between communication theory and practice (45). A reason for that disconnect is that they view theories as too abstract to be applicable in practice. To bridge that gap, then, SCCT, which is variables-driven, is critical to identifying behavioral factors that have been implicated in, say, this evolving health crisis. We, therefore, present three implications of SCCT for the Wuhan government's virus-testing program, in particular, and for health communication strategies, in general.

First, one of the challenges of the Wuhan citywide testing program was mixed messaging, by which clarity of guidelines distributed by governments and agencies was in question. Therefore, the health-communication crisis spawned initially a communication crisis—mixed messaging—that is being resolved through strategies embedded in an evolving pandemic and through tactics informed by the strategies government institutions will adopt. One such mixed messaging emanated from viral infodemic, which was implicated in increases in public depression and anxiety; however, it prodded government agencies into launching an effective health communication strategy to mitigate the negative psychological impact of COVID-19 (46, 47).

Second, rumor control. The rumor mill was rife with uncorroborated information during the initial phases of the pandemic in Wuhan. SCCT has the potential to enable health practitioners understand quickly how rumors, as social sources of influence, lead to message believability and to greater attribution of the outcomes of a crisis to organizational—that is, governmental—responsibility (48). SCCT focuses on crisis response contingent on crisis type. For example, a subjective rumor on virus testing spreads on the internet, leading to a snowball in the transmission of that rumor, complicating government's response to it. How did the Wuhan government respond to it eventually? By adopting a containment strategy of ensuring that the life cycle of the rumor was truncated, the government deprived it of further transmission.

Third, health-communication practitioners can draw upon the predictive strengths of SCCT. Zhao (49) asserts: “The social scientific approach develops predictive frameworks that uncover various crisis variables that determine the crisis communication process. While predictive frameworks have recognized the constitutive role of communication, they have also identified social constructions of crises as *naturally occurring* (emphasis added) phenomena” (p. 112). The keywords “naturally occurring” point to the inevitability of social construction as applied to the health crisis that Wuhan initially singularly experienced, constructed—and managed. It was “a process and a product of collective meaning making and ongoing negotiation through complex interactions among multiple social actors in a particular social setting” (pp. 99–100). Health-communication practitioners, even if blind-sided by the onset of a crisis, can promptly reach for SCCT's predictive elements that offer a road map that facilitates that collective meaning making, particularly through its requirement of an identification of crisis type or its assignment to a cluster that is an initial step in assessing crisis responsibility and recommending a crisis-response strategy (50).

## Conclusion

This study focuses on a citywide virus testing to contain the SARS-CoV-2 crisis. It explores how effectively and efficiently the local government system of Wuhan prevented the crisis by involving and mobilizing its three-tier units: city, districts and communities. The prevention-and-control strategy it employed was primarily informed by SCCT, which posits that because situational differences in organizational crises result in different impacts on an organization's post-crisis reputation, organizations are better served by adopting response strategies most appropriate to their situations. In the case of the government of Wuhan, the virus-testing program, widely administered, could provide an effective platform for responding to public anxieties and uncertainties in a fast-evolving pandemic.

Based on the experiences of the first surge in late January 2020, in the current pandemic, SARS-CoV-2 containment is likely to have effective results by implementing strict control measures to test, trace and isolate new cases. Therefore, in the second surge, the Wuhan government's robust testing of its citizens demonstrated its public-health resolve to protect them from the devastating effects of an impending SARS-CoV-2, following the diagnosis of six additional confirmed cases. Such a community-based approach to controlling COVID-19 is a massive investment that requires not only the mobilization of the local government institutions but also the understanding and cooperation of residents. Testing 6.9 million people in 10 days demonstrated the Wuhan local government's proactive control mechanism to tackle a public scourge in its attempt to create a seeming pandemic-proof city—an approach extended to cities such as Beijing, Shanghai and Xinjiang, in their attempts to contain second outbreak. While many countries are still under lockdowns, Wuhan's crisis is a plausible indication of the fate that might await all SARS-CoV-2-stricken countries post-lockdowns; therefore, the recently implemented Wuhan control mechanism (at the city, district and township levels) is poised to serve as a model to other cities.

## Limitations of the Study and Future Research Directions

This article discusses the implementation of citywide nucleic-acid testing in Wuhan. Because of the impact of the epidemic and restrictions on local blockades and 14-day isolation measures issued by governments across China, the authors could not reach into the depths of pandemic-related operations of Wuhan government departments at city, district and township levels and engage specific communities in ethnographic investigations. Because of that limitation, it is highly recommended that future researchers seek to collate evidence based on local community responses and on challenges residents faced during the lockdown. Such studies will help in designing future crisis-management-response clusters, especially for urban communities. In that way, local governments and their agencies can efficiently and effectively contain similar public-health crises in the future. Public-health scholars may investigate the role of local communities in their collective social responsibility to contain the pandemic effectively, as we have observed that people in various countries did not give it a serious attention until the governments went for partial and complete lockdown.

## Data Availability Statement

The original contributions presented in the study are included in the article/supplementary material; further inquiries can be directed to the corresponding authors.

## Author Contributions

LZ: original draft preparation, data collection, method and analysis, and discussion. HS: developing study, reviewing, and editing. NA: identifying significance of the study, developing literature review, theoretical framework, and editing and formatting. JB: reviewing and editing. CP: developing theoretical framework, critiquing, and copyediting. All authors contributed to the article and approved the submitted version.

## Conflict of Interest

The authors declare that the research was conducted in the absence of any commercial or financial relationships that could be construed as a potential conflict of interest.
